# Quantifying the biomass of parasites to understand their role in aquatic communities

**DOI:** 10.1002/ece3.635

**Published:** 2013-06-11

**Authors:** Jason Lambden, Pieter T J Johnson

**Affiliations:** Ecology and Evolutionary Biology, University of ColoradoRamaley N122, Boulder, Colorado, 80309-0334

**Keywords:** Aquatic ecosystem energetics, energy transfer, food webs, host–parasite interactions, parasite density, trematode, wet mass

## Abstract

By infecting multiple host species and acting as a food resource, parasites can affect food web topography and contribute to ecosystem energy transfer. Owing to the remarkable secondary production of some taxa, parasite biomass – although cryptic – can be comparable to other invertebrate and vertebrate groups. More resolved estimates of parasite biomass are therefore needed to understand parasite interactions, their consequences for host fitness, and potential influences on ecosystem energetics. We developed an approach to quantify the masses of helminth parasites and compared our results with those of biovolume-based approaches. Specifically, we massed larval and adult parasites representing 13 species and five life stages of trematodes and cestodes from snail and amphibian hosts. We used a replicated regression approach to quantify dry mass and compared these values with indirect biovolume estimates to test the validity of density assumptions. Our technique provided precise estimates (*R*^2^ from 0.69 to 0.98) of biomass across a wide range of parasite morphotypes and sizes. Individual parasites ranged in mass from 0.368 ± 0.041 to 320 ± 98.1 μg. Among trematodes, adult parasites tended to be the largest followed by rediae, with nonclonal larval stages (metacercariae and cercariae) as the smallest. Among similar morphotypes, direct estimates of dry mass and the traditional biovolume technique provided generally comparable estimates (although important exceptions also emerged). Finally, we present generalized length-mass regression equations to calculate trematode mass from length measurements, and discuss the most efficient use of limited numbers of parasites. By providing a novel method of directly estimating parasite biomass while also helping to validate more traditional methods involving length-mass conversion, our findings aim to facilitate future investigations into the ecological significance of parasites, particularly with respect to ecosystem energetics. In addition, this novel technique can be applied to a wide range of difficult-to-mass organisms.

## Introduction

Parasites have been historically excluded from most investigations of ecosystem function because they are small, difficult to observe, and assumed to contribute relatively little biomass to ecosystems (Hudson et al. 2006). In recent years, however, several studies have suggested that parasites may be important members of ecological communities (Poulin 1999; Horwitz and Wilcox 2005; Hudson et al. 2006; Lefèvre et al. 2009). For instance, inclusion of parasites into ecological food webs can substantially alter estimates of connectance (Lafferty et al. 2006, 2008; Amundsen et al. 2009; Preston et al. 2013), which has been linked to ecosystem stability (McCann 2000; Allesina and Pascual 2008). Parasites can also affect ecosystems indirectly by altering host behavior or the outcome of species interactions, including competition and predation (Schall 1992; Mouritsen and Poulin 2005; Wood et al. 2007; Lefèvre et al. 2009; Preston and Johnson 2010; Orlofske et al. 2012). In New Jersey streams, for instance, the isopod *Caecidotea communis* plays an important role as a detritus consumer; when infected with the parasite *Acanthocephalus tahlequahensis*, however, consumption decreases dramatically, affecting resource availability for other species (Hernandez and Sukhdeo 2008). Collectively, this evidence suggests that parasites have the potential to play an important role in host population dynamics and overall community structure (Poulin 1999; Mouritsen and Poulin 2002; Wood et al. 2007; Holdo et al. 2009; Lefèvre et al. 2009).

**Figure 1:**
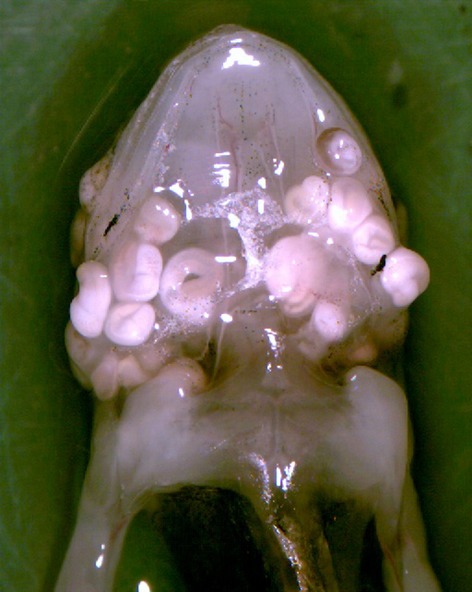
*Clinostomum* metacercariae, a particularly large larval trematode, can comprise a significant portion of an amphibian host's biomass. Image depicts a ventral view of a frog's head with large numbers of metacercariae around the lower mandible.

Recent studies have also challenged the assumption that parasites occupy only a small amount of biomass in ecosystems. While parasites are often small in size, their considerable turnover can facilitate high yearly production (Chu and Dawood 1970). In the open ocean, for instance, viruses may number 3 × 10^8^ per mL of water, which translates to 200 Mt of carbon, making viruses the oceans second largest component of biomass and an important driver of carbon cycling (Suttle 2005; Middelboe 2008). In several well studied Baja California and California salt marshes, total parasite biomass (predominantly larval trematode) was equivalent to total fish biomass and greater than total bird biomass (Kuris et al. 2008). In large part this was due to the remarkably high production of free-living infectious stages, many of which probably fail to find a host and thus become resources for other species (Johnson et al. 2010). Parasites are similarly productive in freshwater ecosystems, where the standing biomass of trematodes has been shown to be second only to mollusks and amphibians and comparable to that of the most abundant insect orders including beetles, damselflies, dragonflies, true insects, and mayflies (Preston et al. 2013) (Fig. 1). Trematodes have dynamic life cycles often involving a definitive host (often a vertebrate), which deposits eggs into aquatic environments that hatch, infect snails, and reproduce asexually to eventually release free-swimming infective cercariae (Olsen 1986). It is these asexually reproducing stages and the cercariae they produce that contribute most notably to the high biomass production of trematodes. While further comparative research is needed, ideally incorporating a wider diversity of both habitat types and parasite groups, the above studies suggest that significant biomass and therefore energy may be moving through parasites via trophic interactions (Kuris et al. 2008; Lafferty et al. 2008; Johnson et al. 2010).

An accurate and more detailed understanding of parasite biomass is essential for understanding the flow of energy through ecosystems with additional potential contributions to investigations of the metabolic theory of ecology (Arneberg et al. 1998; George-Nascimento et al. 2004; Hechinger et al. 2012). Larval trematodes, which often comprise the largest parasite biomass contribution in aquatic ecosystems studied thus far, can directly contribute to energy flow by being consumed by a large variety of predators either as free-living life stages or along with infected hosts (Raffel et al. 2008; Thieltges et al. 2008; Kaplan et al. 2009; Johnson et al. 2010). Because standing biomass calculations often involve extensive extrapolation (i.e., multiplying per capita biomass up to the population or ecosystem scale), they can be highly sensitive to even slight errors in the mass estimates of parasites. Thus far, however, few studies have measured parasite biomass at the population level, and those that have often use an indirect technique that involves multiplying parasite biovolume by an assumed wet mass density of 1.1 g/mL (George-Nascimento et al. 2004; Kuris et al. 2008). The assumed wet mass density of 1.1 g/mL and the additional, implicit, assumption that all parasites have the same percentage water weight remain largely untested, which could have significant consequences for parasite productivity measurements as the mass of individual parasites is often scaled-up many thousands or even millions of times. More accurate measurements may also help to better incorporate parasites into the metabolic theory of ecology, which predicts metabolic rate from body size and temperature in order to explain constraints on life history attributes, population interactions, and ecosystem processes (Brown et al. 2004; Hechinger et al. 2012; Molnár et al. 2012).

Here, we present and validate a method for directly determining the dry mass of parasites spanning a variety of life stages and relative sizes. Using this technique, we measured the dry mass of 10,227 parasites (individually or in aggregate) representing 12 trematode species across four different life stages (rediae, cercariae, meta/mesocercariae, and adults) and one larval cestode. These parasites were collected from subsets of 1423 infected amphibian hosts (16 species) and 1140 infected snail hosts (1 species). From these data, we compared how parasite dry mass changed among life stages (among and within species where possible) and developed general, length-mass regressions for each type of parasite stage. For five of the directly massed parasites, we also estimated wet mass using the biovolume technique and compared these values with direct estimates (i.e., dry mass) to test underlying density assumptions. If density assumptions are correct, we expected the percentage water weight of parasites (difference between the wet mass estimate from indirect technique and dry mass from direct technique) to be consistent with expected values (i.e., percentage water weight estimates from closely related species) and among species and morphotypes. This has important implications in determining the extent to which parasite wet mass can be generally converted to dry mass and used to infer ecological properties such as nutrient deposition via parasites. By developing empirical measurements of parasite dry mass and corresponding length-mass regressions, we aim to facilitate a more accurate understanding of the role of parasites in aquatic ecosystems in both this study and future investigations.

## Methods

### Host collection and necropsy

In 2011, amphibian and snail host species were collected from 86 wetlands in the East Bay region of California (Alameda, Contra Costa, and Santa Clara counties) and 13 US Fish and Wildlife Service National Wildlife Refuges. This included 16 amphibian species and 1562 individual amphibians (see Supplemental Information). All snail parasites as well as *Echinostoma* sp. metacercariae were collected from a subset of 1140 infected *Helisoma trivolvis* (rams horn snail). After collection, host individuals were promptly necropsied. For amphibian hosts, this involved a careful inspection of the cutaneous tissue, musculature, and body cavity for larval and adult macroparasites (Johnson and Hartson 2008; Hartson et al. 2012; Johnson and Hoverman 2012). The larval stages of trematode parasites included rediae and sporocysts (asexually reproducing stages within snail hosts), cercariae (a free-swimming infective stage released from the snail host) and meso/metacercariae (a larval stage derived from a cercariae that has infected an intermediate host or fomite). To isolate cercariae from infected snails, we placed individual snails into 50-mL centrifuge tubes filled with dechlorinated tap water and isolated any emerging cercariae. A subset of infected snails was dissected to collect and mass larval stages within snails (rediae or sporocysts). We identified infections in snails and amphibians to genus and, when possible, to species with molecular analyses (S. A. Orlofske, unpubl. data, Schell 1985). Parasites not identified to species is a result of either a species complex that is difficult to phenotypically distinguish (*Echinostoma* sp., *Alaria* sp., *Fibricola* sp., *Megalodiscus* sp., and *Halipegus* sp.) or because they are a single, unidentified species (*Allassostomoides* sp., *Gorgoderid* sp., *Gorgoderina* sp., *Clinostomum* sp*., Haematoloechus* sp., and *Mesocestoides* sp.).

### Parasite isolation and massing

The technique for quantifying dry mass involved isolating living parasites from host tissue, suspending them in deionized (DI) water, and transfering them onto predried and premassed filters to be redried and remassed. To measure the mass of trematode free-living stages (cercariae), parasites were promptly collected (<4 h postrelease to avoid excessive mass lost to metabolic activity) from field-caught snails (see above) and isolated into ∼100-mL DI water. A known number of cercariae were then carefully pipetted from the DI suspension and vacuum filtered onto a predried (48 h at 60°C) and premassed (Sartorious-CP2P microbalance, Goettingen, Germany) 25-mm filter (Osmonics 0.22 μm nylon mesh). Up to 20 replicates were generated at different sample sizes (e.g., 25, 50, and 100 individual parasites/filter) to create a replicated regression model, which offers a more precise estimate of parasite biomass (see Supplemental Information) (Cottingham et al. 2005). To control for background particles in the filtrate, between 5 and 10 ‘blank’ filters (premassed and prepared in the same way, but without parasites) were made for every 20 filters with parasites. All samples were redried (48 h at 60°C) and massed again using the same microbalance. The mass differences were calculated and corrected using control filters (i.e., the average change in the ‘blank’ filters was added to the mass change in filters with parasites). To control for atmospheric humidity, only five filters were removed from the drying oven at a time and they were kept in a sealed container with Drierite desiccant (W. A. Hammond Drierite Co. LTD, Xenia, OH) until being massed. Meta/mesocercariae and adult parasites were prepared similarly, but the parasites were isolated from amphibian hosts (or external substrates [e.g., snail shells or plastic vials] in the case of *Allassostomoides* metacercariae). Additionally, as these parasites tended to be larger than cercariae, smaller numbers were filtered, depending on the parasite (see Table 1).

**Table 1:** Dry mass results for 16 species/life stages of parasites organized by class/life stage

Class	Lifestage	Species	Mean dry mass (μg)	Standard deviation	Standard error	Number of replicates	Replicated regression		

							Slope	*R*^2^	RMSE
Trematoda	Cercariae	*Echinostoma* sp.	0.368	0.182	0.041	20	0.452	0.70	21.9
Trematoda	Cercariae	*Ribeiroia ondatrae*	0.880	0.255	0.053	23	–	–	–
Trematoda	Mesocercariae	*Alaria* sp.	1.020	0.312	0.140	5	–	–	–
Trematoda	Metacercariae	*Echinostoma* sp.	0.660	0.121	0.046	8	0.662	0.75	20.8
Trematoda	Metacercariae	*Ribeiroia ondatrae*	1.097	0.318	0.071	19	1.126	0.79	15.1
Trematoda	Metacercariae	*Fibricola* sp.	1.570	0.122	0.041	9	1.691	0.89	35.7
Trematoda	Metacercariae	*Manodistomum syntomentera*	2.57	0.310	0.155	3	2.450	0.90	18.7
Trematoda	Metacercariae	*Allassostomoides* sp.	4.400	1.091	0.488	5	–	–	–
Trematoda	Metacercariae	*Gorgoderid* sp.	6.774	2.641	0.591	20	6.443	0.69	61.0
Trematoda	Metacercariae	*Clinostomum* sp.	199.100	78.590	24.850	10	216.9	0.98	103.6
Trematoda	Rediae	*Ribeiroia ondatrae*	4.963	4.226	0.799	27	4.312	0.72	75.4
Trematoda	Adult	*Gorgoderina* sp.	105.604	115.744	57.872	3	–	–	–
Trematoda	Adult	*Megalodiscus* sp.	181.500	99.299	57.330	3	–	–	–
Trematoda	Adult	*Haematoloechus* sp.	320.285	294.366	98.122	9	–	–	–
Trematoda	Adult	*Halipegus* sp.	500.611	64.313	37.131	3	–	–	–
Cestoda	Tetrathyridia	*Mesocestoides* sp.	0.540	0.141	0.044	10	0.591	0.79	20.3

Mean dry mass was determined for all species and life stages along with standard deviation and standard error. For parasite species/life stages used in the replicated regression approach, the slope (which indicates the most precise estimate of mean biomass), root mean square error (RMSE) and *R*^2^ are provided (see Supplemental Information for further detail on sample sizes and ANOVA results).

For rediae and sporocyst stages collected from snails, we made two different measurements of dry mass: (1) the mass of small groups of rediae (using similar methods as described above) and (2) the total parasite dry mass aggregated among all individual rediae or sporocysts relative to snail host mass (excluding shell). This latter approach was incorporated to help facilitate population-level estimates of trematode biomass (which are often inferred from infected snail density) and to help overcome challenges associated with extreme variation in individual rediae/sporocyst size. To determine the total parasite dry mass relative to snail host dry mass, we carefully separated parasite tissue from host tissue (gonads typically) on a 28 mm aluminum weigh boat (predried for 24 h at 60°C and massed using Sartorious-CP2P microbalance). The shell was carefully removed and the parasites were transferred to a separate weigh boat. This involved dissecting the snail gonads, digestive tract, foot and mantle, then placing all detected parasites into a second weigh boat while ensuring that as little host tissue was included as possible. Although, some host tissue could have been included in the parasite weigh boat (and vice versa), we took great care to minimize this possibility. Both sets of weigh boats were redried (24 h) and remassed. The change in mass was corrected using ‘blank’ weigh boats as controls for any background mass changes not due to parasite tissue. This technique was used to determine the relative dry mass of sporocysts (i.e., *Alaria* sp. and *Cephalogonimus* sp.) and rediae (*Allassostomoides* sp., *Echinostoma* sp., *Halipegus* sp., and *R. ondatrae*) (Table 2). For *R. ondatrae* rediae, we also measured the mass of small groups of rediae using the same general method as described for cercariae and meta/mesocercariae. Rediae were isolated from *Helisoma trivolvis* host gonad tissue, suspended in deionized water and filtered onto preweighed and dried nylon filters. Filters were then dried at 60°C for 24 h, remassed and corrected using control filters.

**Table 2:** Proportion of parasite dry mass within a snail host relative to the total dry mass (shell excluded)

Class	Species	Reproductive larval stage	Sample size	% Parasite tissue	Standard error
Trematode	*Alaria* sp.	Sporocyst	8	33.2	3.2
Trematode	*Allassostomoides* sp.	Rediae	9	18.9	1.8
Trematode	*Cephalogonimus* sp.	Sporocyst	5	30.4	2.5
Trematode	*Echinostoma* sp.	Rediae	8	17.7	1.6
Trematode	*Halipegus* sp.	Rediae	7	30.6	2.4
Trematode	*R. ondatrae*	Rediae	9	20.1	1.0

All parasites were taken from *H. trivolvis* hosts and included species with either rediae or sporocyst as the reproductive larval stage. Independently massing both host and parasite tissue offers a measurement of the percentage parasite mass within the snail host.

### Wet mass from biovolume

For five parasite species, biovolume was measured to compare results of the indirect biovolume technique to the direct dry mass method. This included *Ribeiroia ondatrae* metacercariae and rediae, *Echinostoma* sp. metacercariae, *Fibricola* sp. metacercariae, and *Clinostomum* sp. metacercariae. With the exception of *Clinostomum* sp. metacercariae, the volume was measured using Fisherbrand Urisystem decislide (Fisher Scientific, Pittsburgh, PA) of a known depth (0.127 mm). Parasites were placed between the slide and coverslip such that there was no excess space, and photographed using an Olympus DP71 camera (Olympus, Center Valley, PA). Surface area was calculated using the software program ImageJ (Abràmoff et al. 2004), multiplied by the depth to calculate volume, and then converted to wet mass assuming a wet mass density of 1.1 g/mL (George-Nascimento et al. 2004; Kuris et al. 2008). For the largest parasite, *Clinostomum* sp., volume was measured by placing Play-Doh (Hasbro, Pawtucket, RI) spacers of known depth (0.42 mm) between a slide and coverslip.

### Analysis

Two methods were used to assess per-parasite dry mass from these measurements. First, we used replicated regression models constrained through the origin for each species/life stage with multiple, replicate filters containing differing numbers of individual parasites (*Clinostomum* sp. metacercariae, *Echinostoma* sp. cercariae, *Gorgoderid* sp. metacercariae, *Mesocestoides* sp. tetrathyridia (larval cestode), and *R. ondatrae* metacercariae and cercariae). Parasite individual dry mass was then estimated as the slope of the line between the number of parasites per filter and the final (blank corrected) mass of each filter, with root mean square error providing an indicator of precision (Rosner 2011). This analysis was chosen for its ability to provide more power and precision (Cottingham et al. 2005). For each parasite, we also used one-way ANOVA to test whether our estimates of parasite mass (corrected filter mass divided by number of parasites per filter) varied as a function of how many parasites were placed on the filter. For parasites without replicate filters supporting a range of parasites per filter, which often occurred for less common species and all adult parasites, we determined the average change (and standard deviation) in filter dry mass as a function of parasite number.

To assess the density assumptions underlying the biovolume method, we compared the mass estimates for parasites measured using both techniques. The biovolume technique provides an indirect estimate of wet mass whereas the direct method used here measures dry mass; the degree to which they differ is assumed to be the percentage water weight of a parasite. We tested the 1.1 g/mL water weight assumption by comparing the average percentage water weight calculated for our parasites to comparable literature estimates. As few studies have directly investigated this metric, we based our expected percentage water weight on estimates from taxonomically similar groups. Turbellaria (free-living flatworms in the same phylum as trematodes) have a water weight of ∼80% whereas earthworms are approximately 75–90% water by mass (Schmitt 1955 [as cited by Tempel & Westheide 1980], Edwards & Bohlen 1995).

Because the direct biomass method is time and labor intensive and requires large numbers of parasites, we used our data to generate a generalized length-mass regression for metacercariae to facilitate accurate conversions to biomass (provided that the density assumptions hold). This involved converting the directly estimated biomass measurements (using the above technique) of six metacercariae (*Clinostomum* sp., *Echinostoma* sp., *R. ondatrae, Manodistomum* sp., *Allassostomoides* sp., and *Gorgoderid* sp.) to log_10_[μg+1]–transformed values and averaging the length of approximately 10 individuals from each species. The log_10_[μg+1]–transformed biomass values were then regressed on log_10_[mm+1]-transformed length values using a linear function and model fit was evaluated using metrics such as *R*^2^. In addition, we investigated the trade-off between having more parasites per filter or more filter replicates (with fewer parasites per filter). This was done by calculating the coefficient of variation in the mean (CV = SE/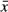) for subset of filters (grouped by species and the number of parasites per filter) (Cyr et al. 1992). We then compared the relative influence of variation in sample size (number of filters), the individual dry mass of the parasite, and the number of parasites per filter on the CV (a measure of precision) using a multiple linear regression approach with STATA statistical software (StataCorp 2005).

## Results

### Direct estimates of parasite biomass

**Figure 2:**
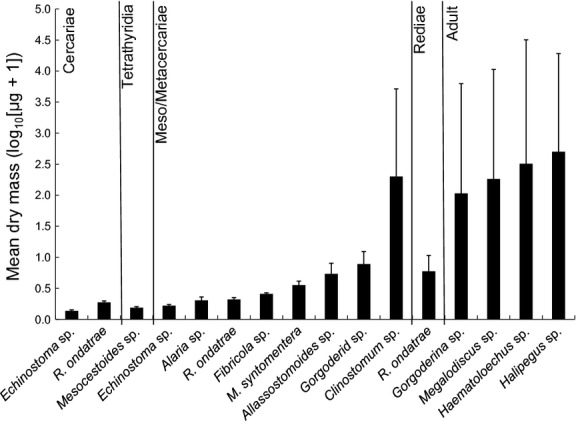
Average dry mass of each species of parasite separated by life stage. Mass values are reported as log_10_[μg+1] transformed values and the error bars indicate log_10_[μg+1] standard error.

The dry mass of individual parasite species and life stages spanned more than three orders of magnitude, with *Echinostoma* sp. cercariae (0.37 ± 0.041 μg [SE]) as the smallest and adult *Halipegus* sp. (500.6 ± 37.13 μg) as the largest (Table 1). In general, adult parasites tended to be the largest (average = 277.0 ± 86.8 μg), whereas cercariae were the smallest (average = 0.62 ± 0.26 μg), with metacercariae (average = 35.77 ± 32.68 μg), mesocercariae (average = 1.295 ± 0.27 μg), larval cestode tetrathyridia (average = 0.54 μg), and rediae (average = 4.96 μg) supporting intermediate values (Fig. 2). However, there was considerable interspecific and intraspecific variation in mass within specific life stages. For instance, the smallest metacercariae, *Echinostoma* sp. (0.37 ± 0.041 μg) were ∼500× smaller than the largest, *Clinostomum* sp. (199.10 ± 24.85 μg). This large size of *Clinostomum* also drives the large average dry mass of metacercariae reported above; excluding *Clinostomum* sp., the average mass of metacercariae declined to 3.10 ± 1.12 μg.

All four life stages investigated demonstrate intraspecific variations in size due to either differences in maturity or stochastic causes. The cercariae size was largely homogeneous, as evidenced by the smaller standard deviation (Table 1), and the metacercariae size variation depended on the relative size of the parasite metacercariae. Smaller metacercariae (*Echinostoma* sp. and *R. ondatrae)* had less variation in size whereas larger metacercariae (*M. syntomentera*, *Gorgoderid* sp., and especially *Clinostomum* sp.) had greater intraspecific variation (Table 1). Finally, the rediae and adults massed all exhibited a relatively large amount of intraspecific size variation (Table 1).

*Ribeiroia ondatrae,* a trematode that causes amphibian limb deformities, was massed at three larval stages, offering information on mass transitions among hosts. The smallest stage was the cercaria (0.880 ± 0.053 μg), which increased in mass by ∼25% after they infected an amphibian to form a metacercaria (1.097 ± 0.071 μg). *Ribeiroia* rediae isolated from snails were the largest larval stage (4.963 ± 0.799 μg), or ∼350% larger than the metacercaria or cercaria.

Measurements of total parasite biomass within a snail host revealed that, across all species measured, the percentage parasite dry mass relative to total dry mass (snail and parasite, not including shell) varied from 7.5% to 46.5% with an average percentage parasite mass of 24.5 ± 1.2%. Species with sporocysts (*Alaria* sp. and *Cephalogonimus* sp.) comprised a greater total fraction of host biomass (average = 31.8 ± 1.4%) than those with rediae (*R. ondatrae, Echinostoma* sp. *Allassostomoides* sp., and *Halipegus* sp.) (average = 21.8 ± 2.9%), even after accounting for variation in snail host size (ANOVA with snail size as a covariate, *F*_2,38_ = 11.918, *P* < 0.0001) (Table 2). Most of the snails measured in this way were relatively large and harbored mature infections that supported active cercariae production (see Supplemental Information). Dividing the total parasite mass of *R. ondatrae* within snail hosts by the directly measured mass of individual redia (4.96 ± 0.799 μg), we estimated a total intrasnail population of rediae ranging from 710 to 2684, which is within the range of what has been observed empirically (P. T. J. Johnson, unpubl. data), although these estimates will likely be sensitive to maturity of the infection within a snail.

**Figure 3:**
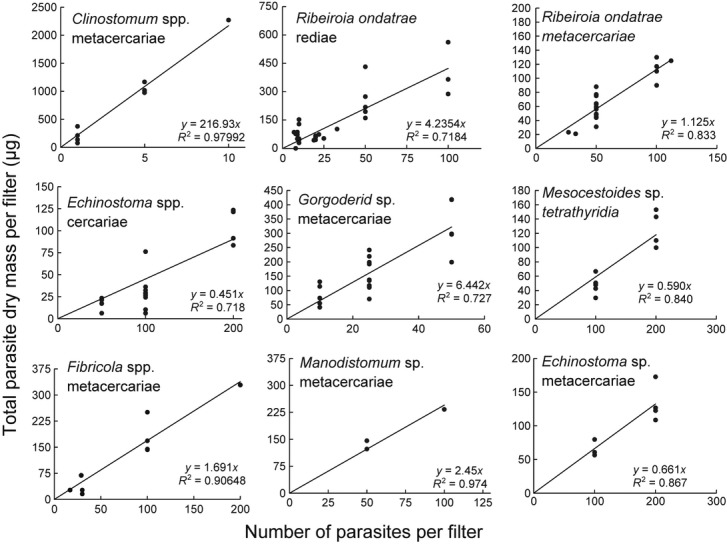
Linear regression for the nine parasite species/life stages for which replicated regression was performed. We compare the number of parasites on a filter to the total corrected dry mass of the filter after drying. Trend lines are constrained through zero, and *R*^2^ values are reported along with the regression equation where slope is equivalent to parasite mass.

On the basis of the replicated regression analysis, we observed a generally robust relationship between the number of parasites per filter and their aggregate mass, with *R*^2^ values ranging from 0.69 to 0.98 (Fig. 3). Groups with high variation included *Echinostoma* metacercariae and rediae, *Gorgoderid* sp. metacercariae and *R. ondatrae* rediae, in part due to the small sizes of individuals and/or considerable intraspecific variation in size. The precision of these replicated regression results was supported by the overall low residual error reflected by the root mean square error (Table 1). For species with filter replicates that had varying numbers of parasites, ANOVA results generally did not find a significant effect of sample size on mass estimate, suggesting that the biomass measurements were consistent across filters with varying numbers of parasites (within species) (Supplemental Information).

### Indirect biovolume estimates

**Figure 4:**
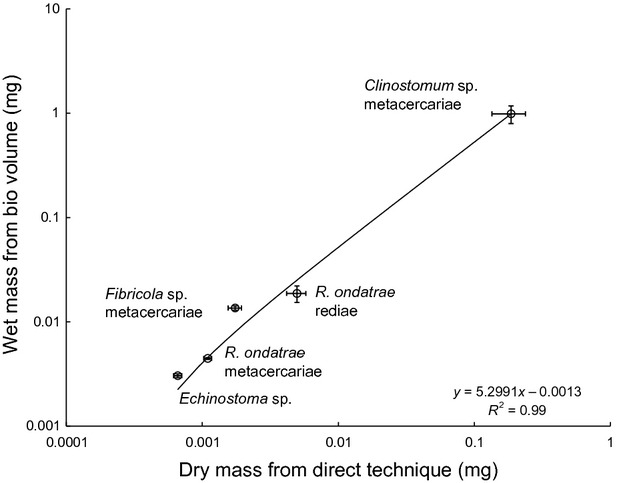
Linear regression using average wet mass results from the biovolume technique and average dry mass from the direct technique. Both techniques were used to mass metacercariae of four trematode species (*R. ondatrae, Clinostomum* sp.*, Fibricola* sp., and *Echinostomum* sp.) and rediae of one species (*R. ondatrae*), which permits estimates of the percentage water weight (see Supplemental Information).

The wet mass measurements of individual parasites derived using biovolume included *Echinostoma* sp. metacercariae (*n* = 12, 3.04 ± 0.13 μg), *R. ondatrae* metacercariae(*n* = 13, 4.45 ± 0.09 μg) and rediae (*n* = 31, 18.70 ± 2.32 μg), *Fibricola* sp. metacercariae (*n* = 8, 13.54 ± 0.64 μg), and *Clinostomum* sp. metacercariae (*n* = 5, 984.49 ± 189.72 μg). Comparing these wet mass estimates to the direct measurements of dry mass (see Supplemental Information), we estimated the percentage water weight of each parasite as 1-(wet mass/dry mass) (see Fig. 4). For *Clinostomum* sp. metacercariae the dry mass, and thus percentage water weight, were calculated for individual parasites, but for the other four parasites percentage water weight is a function of the average wet and dry mass. The average percentage water weight calculated from this difference (79.1 ± 2.4%) corresponds well with the expected range derived from the literature (Supplemental Information). Examining the interspecific variation in water weight, the relationship between parasite wet mass and dry mass was strongly linear (*R*^2^ = 0.99) (Fig. 4); however, much of this pattern was driven by the largest parasite (*Clinostomum* sp.) which, when removed, lowered the overall fit somewhat (*R*^2^ = 0.80).

**Figure 5:**
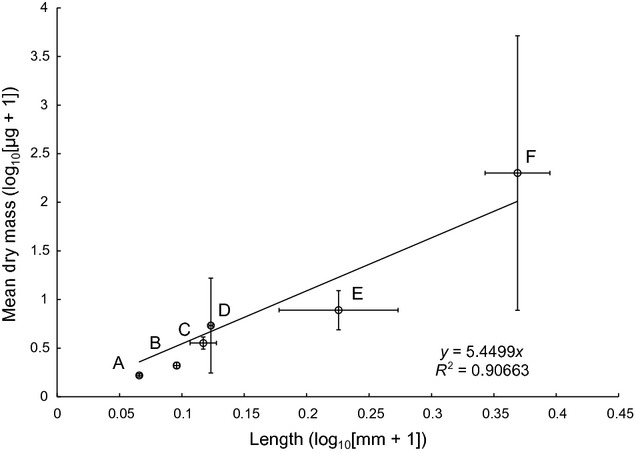
Mean dry mass (log_10_[μg+1]) regressed on length (log_10_[mm+1]) using a linear function constrained through zero for six species of trematode metacercariae. Species of trematode metacercariae included here are *Echinostoma* sp. (A), *R. ondatrae* (B), *Manodistomum* sp. (C), *Allassostomoides* sp. (D), *Gorgoderid* sp. (E), and *Clinostomum* sp. (F). Excluding the larger parasite *Clinostomum*, the slope is reduced (*R*^2^ = 0.80, *y* = 4.301*x*).

Using data from the six parasite species for which we had information on length and mass, we found that a linear log–log relationship constrained through zero captured much of the variation between these two variables (Fig. 5). Parasite length explained 91% of the variation in our empirical dry mass estimates (log_10_[μg+1] = 5.4499 (log_10_[mm+1])). This strong relationship was, to some extent, driven by the largest parasite, *Clinostomum* sp., yet excluding this parasite still yielded a moderately strong relationship (*R*^2^ = 0.80, *y* = 4.301*x*).

**Figure 6:**
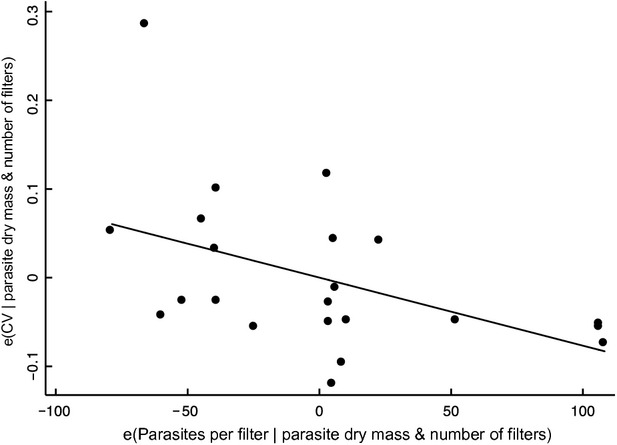
Adjusted variable plot showing the relationship of parasites per filter and the coefficient of variation of the mean adjusting for the number of filter replicates and the mass of the parasite. The slope of −0.00077 (*P* = 0.038) is equivalent to the parasites per filter coefficient observed in a multiple linear regression of CV on parasites per filter, mass of parasites and number of filters (Supplemental Information).

Finally, results of the multiple linear regression analysis suggested that the coefficient of variation estimate was most strongly associated with number of parasites per filter (coefficient = −0.00077, *P* = 0.038), rather than the number of filters used (coefficient = −0.0043, *P* = 0.24) or the mass of the parasite (coefficient = −0.00033, *P* = 0.366) (Supplemental Information). The relationship between the CV and parasites per filter is shown graphically using an adjusted variable plot which regresses the residuals of the CV and number of filters relationship on the parasites per filter and the number of filters relationship. This provides a graphical interpretation of the relationship between the CV and parasites per filter, adjusted for the number of filters (Fig. 6).

## Discussion

The influence of parasites on the flow of materials and energy within communities remains relatively unexplored, yet several reports suggest that parasite biomass within marine and freshwater ecosystems can be considerable (Kuris et al. 2008; Middelboe 2008; Preston et al. 2013). This often-ignored production of biomass has potentially important implications for food web ecology, community ecology, metabolic theory, and ecosystem energetics (Lafferty et al. 2006, 2008; Johnson et al. 2010; Hechinger et al. 2012; Johnson and Hoverman 2012). In order to understand the ways in which parasite biomass affects species interactions and ecosystem properties, it is essential to develop direct and accurate methods of quantifying the biomass of parasites.

Our results, which represent one of the few studies to directly measure the mass of both free-living and parasitic stages of multiple parasite species, provide dry mass estimates for a broad range of parasite species, life stages, and sizes. The use of replicate samples with varying numbers of individual parasites facilitated a replicated regression analysis, which offered more precise estimates of biomass in which the slope is equivalent to the biomass of a single parasite. This was evident by the relatively high *R*^2^ values, indicating that the number of parasites per filter strongly predicted the corrected mass of the filter in a consistent manner. Additionally, the ANOVA results suggested that, with the exception of *Echinostoma* sp., the biomass estimate did not change appreciably as a function of the number of parasites per filter, providing further confidence in these estimates. The *Echinostoma* sp. result could be due to blank filters that had more background particulates in one replicate group, but it could also stem from random chance given the number of tests performed (this result is eliminated when a Bonferroni correction is applied).

We found significant biomass variation not only among species and life stages but also within species. On average, the free-swimming cercarial stage, as the smallest of the larval stages, was 42.5× (3.14× when excluding the large *Clinostomum* sp. metacercariae) smaller than metacercariae and 7.95× smaller than rediae. These results are consistent with the energetic expectation that cercariae should be smaller than the rediae/sporocyst stage from which it emerged. Similarly, many metacercariae are metabolically active at some point and should gain some mass from this process, despite the loss of a mobile tail, in addition to a host-derived increases in biomass stemming from the formation of the cyst wall (Uglem and Larson 1987; Larson et al. 1988; Cho et al. 2008). Adult trematodes are also metabolically and reproductively active, resulting in what is typically a larger biomass than any of the larval stages, as evident in the 10.2× increase in biomass from the metacercariae stage to the adult stage (Fujino et al. 1995).

Within life stages, there was considerable size variation among and within species. The differences among species likely owe to differences in life history and physiological trade-offs. As an example of size differences related to life history, *Magnacauda* sp. cercariae (not measured here) have atypically large tails that help attract predators. These predators then become infected upon consuming the parasite (trophic transmission, Dronen 1973). In addition, size variation among cercariae of different species could result from resource competition. The number of free-swimming parasites produced may result from an evolutionary trade-off between smaller, relative inexpensive cercariae that are shed in much higher numbers, but experience reduced infection success or longevity, relative to larger and more expensive cercariae that are shed in lower numbers, but experience greater longevity or infection success (Thieltges et al. 2008). For instance, snails infected with *Allassostomoides* sp. (which have large cercariae) often shed an order of magnitude fewer cercariae than those infected by smaller cercariae, such as *Alaria* sp. (P. T. J. Johnson, unpubl. data).

Size variations among individuals of the same life stage and species may be due to multiple different causes. Cercariae demonstrated relatively little size variation within species possibly because they typically release from snails when they are mature and experience no additional growth postrelease (Schell 1985). Rediae exhibited considerable variation in size within species, likely due to differences in age, maturity, or possibly caste (Whitfield and Evans 1983; Hechinger et al. 2011). Similarly, adults and metacercariae exhibited more variation in size due to differences in maturity (time postinfection), host suitability, or other stochastic influences. This pattern was much more pronounced among adults and metacercariae of species that exhibit significant postinfection growth (e.g., *Clinostomum* sp.) than for smaller bodied metacercariae (e.g., *R. ondatrae* and *Echinostoma* sp.). For such parasites, future work may provide additional insights into the relative roles of parasite maturity and host suitability in affecting estimates of parasite mass.

We also observed substantial variation in the total parasite dry mass within snail hosts similar to values reported in previous studies. Trematode parasites within snails comprised a range of 7.5–46.5% of the nonshell tissue dry mass, similar to the estimate from estuarine systems of 14–39% (Hechinger et al. 2009). In addition, we found that populations of sporocysts tended to comprise more relative mass than parasites using rediae, even after correcting for snail size. Although, our sample size was limited in terms of the number of species we could include, one possible explanation of this is that rediae, which have a developed digestive system, more actively consume snail tissue compared with sporocysts, which lack a well developed digestive system (Esch et al. 2001). The damage done to the snail host by the rediae could therefore impose an upper limit on the total number or biomass of rediae, with mortality occurring above a certain threshold (Esch et al. 2001). How this pattern persists with inclusion of more parasite species and across other host snail species (e.g., *Lymnaea* and *Physa*) will be an interesting future line of inquiry.

The biovolume technique, which is the most common way researchers have estimated parasite mass previously (George-Nascimento et al. 2004; Kuris et al. 2008), provides an indirect measure of wet mass rather than a direct measure of dry mass, as done here. However, by developing a regression between empirically measured dry mass and the estimated wet mass on the basis of biovolume, we found that, on average, the percentage water weight of parasites (79%) was similar to what we would expect based on morphologically similar free-living taxa (Schmitt 1955 [as cited by Tempel & Westheide 1980], Edwards & Bohlen 1995). This suggests that the wet mass measurements generated by combining parasite size with an assumed parasite wet mass tissue density of 1.1 g/mL are likely reasonable. However, there was considerable variation in the estimated percentage water weight among the parasites examined, ranging between 73.4% and 87.1%, suggesting that caution should be exercised with large extrapolations; a 13.7% difference between the highest and lowest percentage water weights will amount to a large bias when multiplied through by the total parasite population. This difference was particularly pronounced for *Fibricola* sp. (87.1% water weight) and *Clinostomum* sp. (81.0% water weight). These parasites tended to have relatively thin cyst walls and are often more active within the host, possibly contributing to the larger percentage water weight (Chandler 1942).

To facilitate future estimates of parasite dry mass, we provide a length-mass regression for metacercariae and recommendations regarding optimal sample sizes and number of replicates, given limited numbers of parasites and time. The length-mass regression should permit simple calculations of metacercariae dry mass according to the equations specified in the results, although care should be taken in applying this equation to parasites with very different characteristics (e.g., those lacking cyst walls [mesocercariae] or those with very thick encystment properties). On the basis of our results, which illustrate some of the challenges inherent to detecting parasite mass over the ‘noise’ associated with filters and contaminant particulates, we suggest that increasing the number of parasites per filter is generally a better investment (each additional parasite added will decrease the CV [or increase precision] by −0.00077, which can be considerable) than increasing the number of replicate filters (Fig. 6).

## Conclusion

Because parasites are one of the most diverse and speciose groups on earth (Price 1980; Dobson et al. 2005), accurate methods and estimates of their biomass can help to inform studies related to food web ecology, ecosystem energetics, and metabolic theory. Knowing the scaling relationships between parasite body size (biomass) and metabolism, for instance, will provide a valuable addition to the metabolic theory of ecology (George-Nascimento et al. 2004; Poulin 2007; Poulin and George-Nascimento 2007; Hechinger et al. 2012; Molnár et al. 2012). Here, we provided methods for determining parasite mass and regression equations with the aim of facilitating simple determinations of parasite mass in future research. However, to fully explore the applications of parasite biomass, it remains critical to determine the mass of parasites from other species, taxa, and ecosystems.

## Supporting Information

**Table S1.** Detailed description of the parasite sample size per replicate (and number of replicates) along with ANOVA analysis across sample sizes.

**Table S2.** Common and scientific names of the amphibian species sampled and the total number sampled.

**Table S3.** Dry and wet mass comparisons for five parasites, and the percentage water weight determined from the difference in the values.

**Table S4.** Multiple linear regression results table comparing the independent effects of the number of parasites added to each filer and the number of filters massed on the coefficient of variation of the mean (a measure of precision).

**Table S5.** The total parasite mass within *Helisoma* snail hosts.
