# Supplementary material for: Quantifying the biomass of parasites to understand their role in aquatic communities
Source: Ecol Evol. 2013 Jun 11;3(7):2310–21. doi: 10.1002/ece3.635 (PMC3728967; doi:10.1002/ece3.635)
Supplement: Supplementary file 1 [file ece30003-2310-SD1.doc]

Supplemental Information:

S1: Detailed description of the parasite sample size per replicate (and number of replicates) along with ANOVA analysis across sample sizes. Results are stratified by parasite species, life stage and Class. Sample size vales are reported in the format number of parasites/filter (number of filters massed using this sample size). ANOVA results suggest a mean difference for *Echinostoma* sp. cercariae, but if a Bonferroni correction is used to account for multiple tests, alpha becomes 0.017, suggesting this difference could be an artifact.

| **Class** | **Life stage** |  |  | **Anova** | | |
| --- | --- | --- | --- | --- | --- | --- |
|  |  | **Species** | **Sample Size**  **(Replicate Size (#)…)** | **df** | **F** | **P-value** |
| Trematoda | Cercariae | *Echinostoma* sp. | 50(4), 100(11), 200(5), | 2,17 | 0.0005 | 0.70 |
| Trematoda | Cercariae | *Ribeiroia ondatrae* | 100(23) | --- | --- | --- |
| Trematoda | Cercariae | *Magnacauda* sp. |  |  |  |  |
| Cestoda | Tetrathyridia | *Mesocestoides* sp. | 100(6), 200(4) | 1,8 | 0.0006 | 0.79 |
| Trematoda | Mesocercariae | *Alaria* sp. | 19(1), 63(1), 100(3) | --- | --- | --- |
|  |  |  |  |  |  |  |
| Trematoda | Metacercariae | *Echinostoma* sp. | 100(4), 200(4) | 1,5 | 0.0007 | 0.75 |
| Trematoda | Metacercariae | *Ribeiroia. ondatrae* | 27(1), 33(1), 50(12), 100(4), 112(1) | 1,15 | 0.0011 | 0.83 |
| Trematoda | Metacercariae | *Fibricola* sp. | 17(1), 29(1), 30(2), 100(4), 200(1) | 2,5 | 0.0017 | 0.89 |
| Trematoda | Metacercariae | *Manodistomum* sp. | 50(2), 100(1) | --- | 0.0025 | 0.90 |
| Trematoda | Metacercariae | *Allassostomoides* sp. | 30(5) | --- | --- | --- |
| Trematoda | Metacercariae | *Gorgoderid* sp. | 10(5), 25(10), 50(5) | 2,17 | 0.0064 | 0.69 |
| Trematoda | Metacercariae | *Clinostomum* sp. | 1(5), 5(4), 10(1) | 2,7 | 0.2169 | 0.98 |
| Trematoda | Rediae | *Ribeiroia ondatrae* | 7(1), 8(1), 9(3), 10(6), 19(1), 20(3), 22(1), 25(2), 33(1), 50(5), 100(3) | 2,13 | 0.0042 | 0.71 |
| Trematoda | Adult | *Gorgoderina* sp. | 1(2), 3(1) | --- | --- | --- |
| Trematoda |  | *Megalodiscus* sp. | 1(2), 5(1) | --- | --- | --- |
| Trematoda |  | *Haematoloechus* sp. | 3(6), 5(3) | --- | --- | --- |

S2: Table including common and scientific names of the amphibian species sampled and the total number sampled.

| **Species Sampled** | | **Number Sampled** |
| --- | --- | --- |
| **Common Name** | **Scientific Name** |
| American toad | *Anaxyrus americanus* | 42 |
| Western toad | *A. boreas* | 154 |
| Southern toad | *A. terrestris* | 2 |
| Grey tree frog | *Hyla versicolor* | 31 |
| Green frog | *Lithobates clamitans* | 28 |
| American bullfrog | L. catesbeianus | 206 |
| Chiricahua leopard frog | *L chiricahuensis* | 15 |
| Northern leopard frog | *L. pipiens* | 83 |
| Mink frog | *L. septentrionalis* | 27 |
| Southern leopard frog | *L. sphenocephalus* | 29 |
| Wood frog | *L. sylvaticus* | 2 |
| Boreal chorus frog | *Pseudacris maculata* | 45 |
| Pacific chorus frog | *P. regilla* | 691 |
| Northern red-legged frog | Rana aurora | 45 |
| Rough-skinned newt | *Taricha granulosa* | 6 |
| California newt | *T. torosa* | 156 |

S3: Table including dry and wet mass comparisons for five parasites, and the percentage water weight determined from the difference in the values.

| **Parasite** | **Stage** | **Dry Mass (µg)** | **Dry Mass SE** | **Wet Mass (µg)** | **Wet Mass SE** | **Water Weight (%)** |
| --- | --- | --- | --- | --- | --- | --- |
| *R. ondatrae* | Rediae | 4.96 | 0.799 | 18.67 | 3.35 | 73 |
| *Echinostoma* sp. | Metacercariae | 0.660 | 0.121 | 3.04 | 0.124 | 78 |
| *R. ondatrae* | Metacercariae | 1.10 | 0.073 | 4.45 | 0.088 | 75 |
| *Fibricola* sp. | Metacercariae | 1.74 | 0.198 | 13.5 | 0.640 | 87 |
| *Clinostomum* sp. | Metacercariae | 186.0 | 51.3 | 984.5 | 189.7 | 81 |

S4. Multiple linear regression results table comparing the independent effects of the number of parasites added to each filer and the number of filters massed on the coefficient of variation of the mean (a measure of precision).

| **Source** | **SS** | **df** | **MS** |  |  |  |  |  | Number of obs: | 22 |  |
| --- | --- | --- | --- | --- | --- | --- | --- | --- | --- | --- | --- |
| Model | 0.040 | 3 | 0.013 |  |  |  |  |  | F (3,18): | 1.85 |  |
| Residual | 0.130 | 18 | 0.007 |  |  |  |  |  | Prob > F: | 0.174 |  |
| Total | 0.170 | 21 | 0.008 |  |  |  |  |  | R^2^: | 0.236 |  |
|  |  |  |  |  |  |  |  |  | RMSE: | 0.085 |  |

| **CV_x̅_** | **Coef** | **Std. Error** | **t-statistic** | **P-value** | **95% Confidence Interval** | |
| --- | --- | --- | --- | --- | --- | --- |
| Parasites per Filter | -0.00077 | 0.000343 | -2.24 | 0.038 | -0.00149 | -0.000047 |
| Parasite mass | -0.000336 | 0.000362 | -0.93 | 0.366 | -0.00109 | 0.000425 |
| Number of Filters | -0.00435 | 0.00357 | -1.22 | 0.238 | -0.01188 | 0.00314 |
| Constant | 0.246 | 0.0473 | 5.20 | <0.000 | 0.1465 | 0.34519 |

S5. Table showing the total parasite mass within *Helisoma* snail hosts. The snail size indicates the length of the snail shell from the lip of the opening to the far edge, parallel to the opening. The parasites were extracted using the technique described in the methods, and the parasite and snail dry mass were used to calculate the percentage of total parasite mass.

| **Snail number** | **Snail size (mm)** | **State/site** | **Parasite species** | **Principle asexually reproductive stage** | **Infection strength (%)** | **Snail dry mass (mg)** | **Parasite dry mass (mg)** |
| --- | --- | --- | --- | --- | --- | --- | --- |
| 1 | 12.41 | Oregon/P156 | *Ribeiroia ondatrae* | Rediae | 60 | 28.4 | 9.7 |
| 2 | 11.03 | Oregon/P156 | Unknown | Sporocyst | 60 | 15.0 | 5.9 |
| 3 | 13.61 | Oregon/P156 | *Allassostomoides* sp. | Rediae | 50 | 23.3 | 7.3 |
| 4 | 14.66 | Oregon/P156 | *Ribeiroia ondatrae* | Rediae | 40 | 40.6 | 7.8 |
| 5 | 12.95 | Oregon/P156 | *Ribeiroia ondatrae* | Rediae | 75 | 25.4 | 6.7 |
| 6 | 11.38 | Oregon/P156 | *Allassostomoides* sp. | Rediae | 25 | 21.2 | 3.5 |
| 7 | 14.14 | Oregon/P156 | *Ribeiroia ondatrae* | Rediae | 50 | 36.0 | 6.4 |
| 8 | --- | Oregon/P156 | *Allassostomoides* sp. | Rediae | --- | 31.7 | 2.7 |
| 9 | 13.46 | Oregon/P156 | *Ribeiroia ondatrae* | Rediae | 60 | 44.4 | 12.1 |
| 10 | 12.71 | Oregon/P156 | *Allassostomoides* sp. | Rediae | 90 | 24.0 | 5.2 |
| 11 | --- | Oregon/P156 | *Ribeiroia ondatrae* | Rediae | --- | 52.5 | 13.3 |
| 12 | --- | --- | *Cephalogonimus* sp. | Sporocyst | --- | 54.1 | 17.0 |
| 13 | 23.6 | Minnesota/EDP | *Echinostoma* sp. | Rediae | 70 | 56.7 | 11.4 |
| 14 | --- | Oregon/P156 | *Allassostomoides* sp. | Rediae | --- | 37.4 | 13.1 |
| 15 | --- | Oregon/P156 | *Allassostomoides* sp. | Rediae | --- | 34.0 | 8.3 |
| 16 | 15.05 | California/TGIF | *Ribeiroia ondatrae* | Rediae | 65 | 33.7 | 8.0 |
| 17 | --- | Oregon/P156 | *Ribeiroia ondatrae* | Rediae | --- | 38.5 | 9.7 |
| 18 | --- | California | *Alaria* sp. | Sporocyst | --- | 8.4 | 4.5 |
| 19 | --- | Minnesota/EDP | *Echinostoma* sp. | Rediae | --- | 60.1 | 18.6 |
| 20 | 16.82 | Minnesota/EDP | *Echinostoma* sp. | Rediae | 30 | 89.8 | 23.8 |
| 21 | 14.47 | Oregon/P156 | *Allassostomoides* sp. | Rediae | 30 | 15.8 | 4.2 |
| 22 | 12.69 | Oregon/P156 | *Ribeiroia ondatrae* | Rediae | 40 | 14.0 | 3.9 |
| 23 | 15.02 | Oregon/MHCC | *Echinostoma* sp. or  *Ribeiroia ondatrae* | Rediae | 10 | 57.7 | 10.3 |
| 24 | 14.22 | Oregon/P156 | *Allassostomoides* sp. | Rediae | 60 | 16.3 | 4.4 |
| 25 | 18.05 | Oregon/MHCC | *Echinostoma* sp. or  *Ribeiroia ondatrae* | Rediae | 30 | 74.0 | 14.4 |
| 26 | 14.87 | California/TGIF | *Allassostomoides* sp. | Rediae | 60 | 49.0 | 11.9 |
| 27 | 14.1 | California | *Alaria* sp. | Sporocyst | 90 | 14.4 | 6.9 |
| 28 | 10.56 | California | *Alaria* sp. | Sporocyst | 40 | 23.1 | 4.2 |
| 29 | 17.83 | California | *Echinostoma* sp. | Rediae | 40 | 46.6 | 8.4 |
| 30 | 12.45 | California | *Echinostoma* sp. | Rediae | 40 | 36.2 | 3.9 |
| 31 | 15.16 | California | *Echinostoma* sp. | Rediae | 50 | 19.9 | 6.0 |
| 32 | 18.83 | California | *Echinostoma* sp. | Rediae | 70 | 65.3 | 13.1 |
| 33 | 11.89 | California | *Cephalogonimus* sp. | Sporocyst | 90 | 13.6 | 8.1 |
| 34 | 11.25 | California | *Alaria* sp. | Sporocyst | 100 | 8.2 | 4.2 |
| 35 | 13.23 | California | *Alaria* sp. | Sporocyst | 100 | 16.6 | 10.5 |
| 36 | 12.7 | California/Murky Bullfrog | *Halipegus* sp. | Rediae | --- | 13.5 | 4.6 |
| 37 | 10.26 | California/Bart’s Pond | *Halipegus* sp. | Rediae | --- | 13.9 | 4.6 |
| 38 | 11.86 | California/Bart’s Pond | *Alaria* sp. | Sporocyst | --- | 13.0 | 5.0 |
| 39 | 12.95 | California/Bart’s Pond | *Halipegus* sp. | Rediae | --- | 21.7 | 10.2 |
| 40 | 11.48 | California/Bart’s Pond | *Halipegus* sp. | Rediae | --- | 19.9 | 6.8 |
| 41 | 12.4 | California/Bart’s Pond | *Halipegus* sp. | Rediae | --- | 15.0 | 8.8 |
| 42 | 9.63 | California/Bart’s Pond | *Alaria* sp. | Sporocyst | --- | 5.4 | 3.2 |
| 43 | 12.16 | California/Bart’s Pond | Unknown | Sporocyst | --- | 12.5 | 8.9 |
| 44 | --- | California/Hidden Pond | *Alaria* sp. | Sporocyst | --- | 8.0 | 6.9 |
| 45 | 12.86 | California/Bart’s Pond | *Halipegus* sp. | Rediae | --- | 13.2 | 9.2 |
| 46 | 12.41 | California/Bart’s Pond | *Halipegus* sp. | Rediae | --- | 20.6 | 8.0 |
| 47 | 17.00 | California/Garin | *Echinostoma* sp. | Rediae | --- | 65.0 | 11.8 |
| 48 | 12.34 | California/Garin | *Cephalogonimus* sp. | Sporocyst | --- | 22.3 | 7.5 |
| 49 | 14.66 | Minnesota/EDP | *Cephalogonimus* sp. | Sporocyst | --- | 24.2 | 11.8 |
| 50 | 13.17 | California/Garin | *Cephalogonimus* sp. | Sporocyst | --- | 29.8 | 14.6 |
